# Preliminary response to Tislelizumab plus chemotherapy drugs in patient with periampullary carcinoma: a report of one case and a literature review

**DOI:** 10.3389/fimmu.2024.1433235

**Published:** 2024-07-08

**Authors:** Chuanyun Tang, Yijie Kong, Lifan Xu, Chongxu Duan, Xiaowei Fu, Lu Fang, Bo Liang

**Affiliations:** ^1^ Department of General Surgery, The Second Affiliated Hospital of Nanchang University, Nanchang, Jiangxi, China; ^2^ The First Clinical Medical College of Nanchang University, Nanchang, Jiangxi, China; ^3^ Queen Mary School, Jiangxi Medical College of Nanchang University, Nanchang, Jiangxi, China

**Keywords:** periampullary carcinoma, immunotherapy, tislelizumab, chemotherapy, PB-type

## Abstract

Periampullary carcinoma is a malignant gastrointestinal tumor originating from the head of the pancreas, distal bile duct, duodenum, or the ampulla of Vater. Currently, surgery remains the primary treatment option, yet the postoperative recurrence rate remains high. Chemotherapy is the main approach for controlling postoperative recurrence. Histologically, periampullary carcinoma is categorized into two types: intestinal (IN) and pancreaticobiliary (PB) subtype. Each subtype requires different therapeutic approaches, with the PB type primarily treated with gemcitabine and the IN type with 5-FU. Despite these options, patient outcomes are still unsatisfactory. In recent years, the feasibility of immunotherapy in tumor treatment has been increasingly evidenced, although research on its efficacy in periampullary carcinoma treatment is still limited. In this report, we present a case of a periampullary carcinoma patient who experienced recurrence and metastasis after undergoing radical pancreatoduodenectomy and receiving gemcitabine-based chemotherapy post-surgery. Through next-generation sequencing (NGS), we identified high expression levels of programmed cell death-ligand 1 (PD-L1) with a combined positive score (CPS) of 35, high tumor mutation burden (TMB-H), and high microsatellite instability (MSI-H) in this patient. Therefore, we implemented a combination therapy using Tislelizumab and chemotherapy. According to the latest follow-up, the tumors are effectively controlled. Our utilization of immunotherapy combined with chemotherapy holds significant implication for the treatment of periampullary carcinoma.

## 1 Introduction

Periampullary carcinoma is a malignant tumor that originates approximately 2.0 cm from the ampulla of Vater, which can influence to the pancreatic head, the ampulla of Vater, the distal common bile duct and duodenum (1). The majority of these tumors are adenocarcinomas. Currently, surgical resection remains the main therapy option to improve long-term survival and is practicable in approximately 50% of ampullary cancer cases (2). Nonetheless, the overall survival time is still limited due to the high risk of recurrence and metastasis (3). Therefore, adjuvant therapy (AT), such as chemotherapy, is recommended for improving the long-term survival (4).

At present, immunotherapy has emerged as a promising clinical strategy for treatment. In addition, immunotherapy has been incorporated into clinical guidelines for various cancers, including non-small cell lung cancer, esophageal cancer and colorectal cancer (5–7). The assessment of tumor mutation burden (TMB), microsatellite instability (MSI), deficiency of mismatch repair (dMMR), and the expression of PD-L1 are important criteria for determining the individual suitability of immunotherapy (8). However, the prognosis of immunotherapy for patients with periampullary carcinoma remains limited.

Here, we report our experience in treating a periampullary carcinoma patient with high PD-L1 expression, high tumor mutation burden (TMB-H) and high microsatellite instability (MSI-H), who received a combination of Tislelizumab and chemotherapy. The patient demonstrated positive therapeutic outcomes and exhibited acceptable treatment tolerance. By providing comprehensive clinical evaluations and relevant patient histories, we aim to contribute valuable insights into the application of immunotherapy in periampullary carcinoma.

## 2 Case presentation

**Figure 1:**
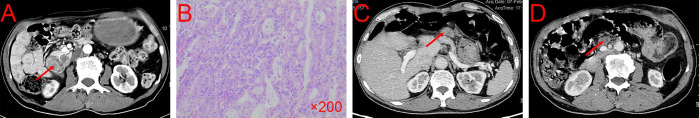
CECT and pathological result of the patient before and after surgery. **(A)** Tumor condition at first CECT scan. **(B)** The results of pathological biopsy. **(C, D)** Tumor conditions after six cycles of gemcitabine + capecitabine. CECT, contrast-enhanced computed tomography.

A 66-year-old male sought medical attention in May 2022 for abdominal pain. Physical examination revealed no yellow pigmentation of the skin or sclera and no abdominal tenderness. Laboratory tests showed: WBC 9.73×10^9^/L, Hb 75g/L, PLT 393×10^9^/L, ALB 38.7g/L, TBIL 7.14μmol/L, ALT 13.78 U/L, ALT 10.36 U/L, ALP 104.6 U/L, γ-GT 13.26 U/L. Cancer antigen 125 (CA125) 84.2U/ml (normal range, 0–35 U/mL), carbohydrate antigen 19–9 (CA19–9) 23.62 U/mL (normal range, 0–37 U/mL), and carcinoembryonic antigen (CEA) 84.20 U/mL (normal range, 0–5U/mL). Contrast-enhanced computed tomography (CECT) of the abdomen showed an occupying area in the duodenal papilla with gastric retention (**Figure 1A**). Preoperative duodenoscopic pathological biopsy confirmed the presence of moderately differentiated adenocarcinoma of the duodenal mucosa.

On May 30, 2022, the patient underwent radical pancreaticoduodenectomy and recovered well without any complications. The postoperative pathological report confirmed a medium-poorly differentiated adenocarcinoma of the duodenum, classified as the PB type (**Figure 1B**). Metastasis was found in 4 of 15 lymph nodes. The patient received six cycles of gemcitabine (1.6g/d, d1, d8, d15, q4w) + capecitabine (2g/d, d1-d21, q4w) as postoperative conventional chemotherapy from August 2022 to November 2022.

**Figure 2:**
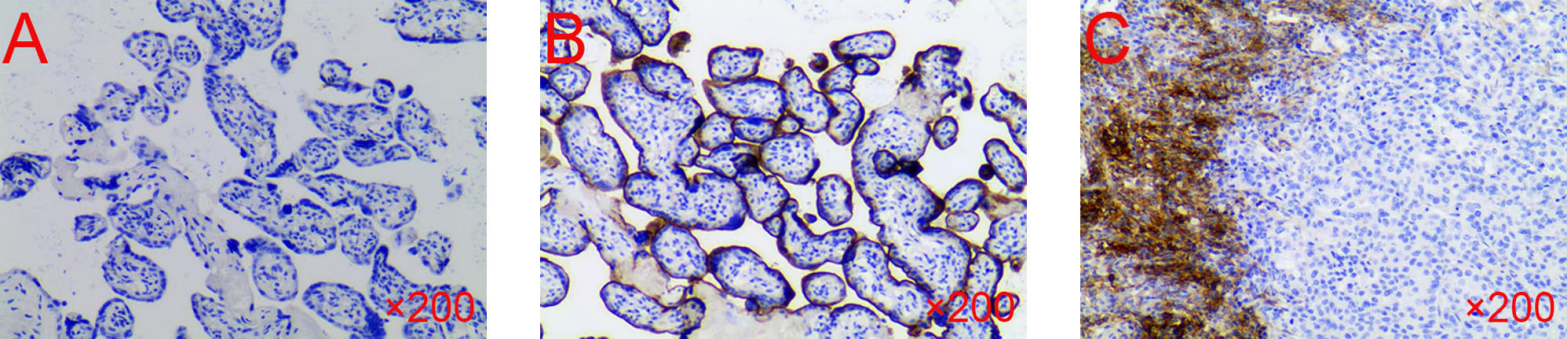
The IHC of the patient’s sample. **(A)** Negative control. **(B)** Positive control. **(C)** The test result of the patient. IHC, immunohistochemistry.

In February 2023, the patient returned to the hospital for reexamination, and the patient had any discomfort and no obvious abnormality on physical examination. However, the laboratory tests showed: CEA 6.57 U/mL (normal range, 0–5U/mL), CA125 9.9U/ml (normal range, 0–35 U/mL), and CA19–9 20.66 U/mL (normal range, 0–37 U/mL). The CECT showed enhancement nodules near the mesentery in the surgical area (**Figures 1C, D**). Due to the suboptimal response to chemotherapy, we performed next-generation sequencing (NGS) on the patient which showed ARID1A, MLH1, PTEN, RAD50, SMARCA4 were mutated and high tumor mutation burden (TMB=30.0 mutations/Mb, normal range<4.5 mutations/Mb) and high microsatellite instability (MSI-H). Furthermore, immunohistochemistry (IHC) demonstrated high expression of PD-L1 (CPS=35, TPS<1%, DAKO 22C3) (**Figures 2A–C**).

**Figure 3:**
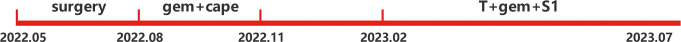
Timeline of the patient’s treatment progress.

**Figure 4:**
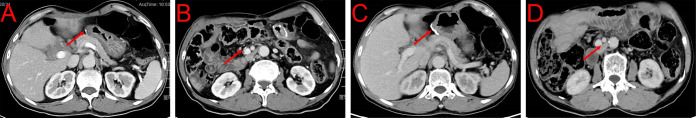
CECT after immunotherapy combined with chemotherapy. **(A, B)** Tumor condition after six cycles of gemcitabine + S-1 + Tislelizumab in May 2023. **(C, D)** Tumor condition after six cycles of gemcitabine + S-1 + Tislelizumab in January 2024.

Based on these results, we decided to administrate chemotherapy combined with immunotherapy for the patient. Between February 2023 and July 2023, the patient received six cycles of gemcitabine (1.6g/d, d1, d8, q3w) + S-1 (100mg/d, d1-d14, q3w) + Tislelizumab (200mg/d, d1, q3w) (**Figure 3**). During the medication, the patient did not have any discomfort and no drug side effects were noted. As of May 2023, CECT indicated that the patient’s tumors shrank 79.5% (from 26.30 cm^3^ to 5.40 cm^3^, PR) (**Figures 4A, B**). The latest CECT report in January 2024 showed the patient was in stable condition, which indicated complete response (CR) (**Figures 4C, D**).

## 3 Discussion

At present, surgical resection is the first choice for the periampullary carcinoma treatment (9, 10), with the postoperative survival rates varying among periampullary carcinoma subtypes at different anatomical locations. Adjuvant chemotherapy is commonly used to improve long-term survival (9, 11), as the general 5-year survival rates for different subtypes of ampullary carcinoma after surgery are between 33% to 68%. Based on heterogeneous mucosal origin, periampullary cancer can be divided into intestinal (IN) and pancreaticobiliary (PB) subtype. The PB type is associated with significantly worse outcomes, with a median overall survival (OS) of 16.1 months compared to 115.5 months for the IN type according to the study of Chang D.K et al. (p <0.001) (12). Different chemotherapy regimens are often used for each subtype, with 5-FU based regimens used for IN type and gemcitabine-based regimens used for PB type (10, 13). Although it is generally accepted that chemotherapy can benefit patients after surgery, whether the utilization of chemotherapy is solidly necessary still remains controversies because of the obvious heterogeneity of the therapeutic effectiveness among patients.

In recent years, the introduction of tumor immunotherapies has revolutionized the cancer treatment landscape. For example, some PD-1 inhibitors, such as Tislelizumab, are being tested its efficacy in clinical trials. In a phase 1/2 trial of Tislelizumab involving 251 Chinese patients with advanced-stage solid tumors who had previously failed anti-tumor treatment, 18% of the cohorts achieved a confirmed response. Notably, patients with nasopharyngeal carcinoma (81%), non-small cell lung cancer (54%), renal cell carcinoma (52%), MSI-H/dMMR solid tumors (50%), and hepatocellular carcinoma (50%) exhibited a favorable clinical benefit rate (CBR ≥50%, defined as the sum of complete response, partial response, and stable disease) (14). In another study involving 15 patients with recurrent or metastatic oral squamous cell carcinoma demonstrated a 40% partial response following treatment with a combination of Tislelizumab and nimotuzumab (15). Furthermore, a study focused on contrasting the effectiveness of Tislelizumab versus chemotherapy in patients with advanced or metastatic esophageal squamous cell carcinoma reported a higher overall response rate in Tislelizumab treatment of 20.9% compared with the chemotherapy of 9.8% (16).

The cellular origin of periampullary carcinoma of PB type may be pancreatic or biliary duct cells. Although immunotherapy has shown limited progress in pancreatic cancer, it has achieved some advancements in biliary tract tumors. Recent large-scale clinical trials investigating the combination of chemotherapy with immunotherapy in biliary cancer have yielded promising results. For instance, the KEYNOTE-966 trial, which involved 1564 patients with locally advanced or metastatic biliary cancer worldwide, demonstrated that the addition of pembrolizumab to gemcitabine and cisplatin significantly improved overall survival (17). Similarly, the TOPAZ-1 study, which enrolled 685 patients with advanced biliary tract cancer, reported that the addition of durvalumab to gemcitabine and cisplatin led to a significant improvement in overall survival compared to the placebo group (18). Furthermore, research conducted by Do-Youn Oh et al. revealed that combining gemcitabine and cisplatin with durvalumab may confer a survival benefit compared to either treatment modality alone (19). These findings underscore the potential synergistic effects of chemotherapy and immunotherapy in improving outcomes for patients with biliary cancer.

According to current clinical studies, immunotherapy has also shown therapeutic efficacy in some cases of periampullary carcinoma. For instance, a case reported by Pothuri et al. described a 59-year-old female who was diagnosed as Lynch’s syndrome and moderately differentiated ampullary adenocarcinoma of PB type with metastasis of adjacent lymph nodes. The patient was determined as MSI-H, TMB-H as well as dMMR type with high PD-L1 expression by the results of NGS and IHC. The patient received three-week administration of four consecutive cycles of nivolumab (1mg/kg) combined with Ipilimumab (3mg/kg) before surgical resection, resulting in a complete pathological response and tumor downstaging from 7.5cm to 5.2cm, which created a favorable prerequisite for further resection. Therefore, the team performed pancreatoduodenectomy on the patient, and the postoperative pathology showed a complete response (20). Another case reported by Wang et al. described a 45-year-old female with advanced ampullary squamous cell carcinoma with lymph node metastasis and high expression of serological CEA (29.43 ng/ml). Initially, the patient received a cycle of chemotherapy with the combination of albumin paclitaxel and cisplatin, but the effect was poorly manifested. Based on patient’s IHC results with a high expression level of PD-L1 in tumor cells (50%) and immune cells (3%), three cycles of chemotherapy combined with immunotherapy (sintilimab 200 mg IVGTT D1) were decided to perform, resulting in a partial response with decreased tumor size and CEA level (11). Coincidentally, Hayley H et al. reported a 75-year-old female with recurrent ampullary carcinoma after receiving surgical resection and postoperative chemotherapy. Based on her genetic detection result of microsatellite stable (MSS) and a high PD-L1 expression level of 35%, the patient began to use pembrolizumab (2mg/kg, every three weeks). After 19 cycles, the patient’s condition was stabilized. Noteworthily, after eight weeks of suspension of immunotherapy, the patient’s condition worsened, but subsequent restart of immunotherapy returned her condition to a stable state (21). Our case report and the above three case reports from other authors all demonstrate the successful application of immunotherapy (combined with chemotherapy) in patients with ampullary cancer. In summary of the cases above, they have all achieved stable condition and even complete pathological response after receiving immunotherapy, which shows a huge potential of immunotherapy for the treatment of periampullary carcinoma in the future. Meanwhile, by considering the patients’ conditions such as mutation load, microsatellite stability and PD-L1 expression through NGS and IHC, the efficacy and prognosis of immunotherapy can be more precisely forecasted, which is remarkable for deciding whether to use immunotherapy.

However, disadvantages of immunotherapy still exist. Periampullary carcinoma patients may experience some immune-related adverse events (irAEs) following the use of immune checkpoint inhibitors (ICIs), such as PD-1 monoclonal antibody Tislelizumab. Common irAEs include immune-related pneumonia, hepatitis, cholangitis, and myocarditis (22). Additionally, immunotherapy resistance has been observed especially for patients with comorbidity disease (23, 24), highlighting the need for monitoring and further research on immunotherapy resistance.

This case report has limitation. Due to various complex factors, it is challenging to determine whether the treatment effects resulted from immunotherapy combined with chemotherapy or from immunotherapy alone. Moreover, there is limited number of reports on immunotherapy for periampullary carcinoma to corroborate our results. Therefore, further basic experiments are needed to explore the underlying molecular mechanisms.

## 4 Conclusions

The case we report suggests that immunotherapy combined with chemotherapy is a safe and effective treatment option for periampullary carcinoma patient, and may be more useful for those with high expression of immunotherapy markers (PD-L1, TMB, MSI and MMR).

## Data availability statement

The original contributions presented in the study are included in the article/supplementary material. Further inquiries can be directed to the corresponding authors.

## Ethics statement

Written informed consent was obtained from the individual(s) for the publication of any potentially identifiable images or data included in this article.

## Author contributions

CT: Writing – original draft, Data curation. YK: Writing – original draft, Data curation. LX: Writing – original draft, Data curation. CD: Writing – original draft, Data curation. XF: Writing – review & editing. LF: Writing – review & editing. BL: Writing – review & editing, Project administration, Funding acquisition, Conceptualization.
